# Malignancy rates in children, young people and adults with JIA: an observational study using CPRD Aurum

**DOI:** 10.1093/rheumatology/keag291

**Published:** 2026-06-04

**Authors:** Lianne Kearsley-Fleet, Samuel W D Merriel, Natasha Shaw, Jasmine Leslie, Michelle Johnson, Lucy R Wedderburn, Kimme L Hyrich, Jenny H Humphreys

**Affiliations:** Division of Musculoskeletal Medicine and Dermatological Sciences, Centre for Epidemiology Versus Arthritis, University of Manchester, Manchester Academic Health Sciences Centre, Manchester, UK; Centre for Primary Care & Health Services Research, School of Health Sciences, The University of Manchester, Manchester, UK; Division of Musculoskeletal Medicine and Dermatological Sciences, Centre for Epidemiology Versus Arthritis, University of Manchester, Manchester Academic Health Sciences Centre, Manchester, UK; Division of Musculoskeletal Medicine and Dermatological Sciences, Centre for Epidemiology Versus Arthritis, University of Manchester, Manchester Academic Health Sciences Centre, Manchester, UK; Division of Musculoskeletal Medicine and Dermatological Sciences, Centre for Epidemiology Versus Arthritis, University of Manchester, Manchester Academic Health Sciences Centre, Manchester, UK; Infection, Immunity and Inflammation Research and Teaching Department, UCL Great Ormond Street Institute of Child Health, London, UK; NIHR Biomedical Research Centre at Great Ormond Street Hospital, London, UK; Centre for Adolescent Rheumatology at UCL, UCL Hospital and Great Ormond Street Hospital, London, UK; Division of Musculoskeletal Medicine and Dermatological Sciences, Centre for Epidemiology Versus Arthritis, University of Manchester, Manchester Academic Health Sciences Centre, Manchester, UK; NIHR Manchester Biomedical Research Centre, Manchester University NHS Foundation Trust, Manchester, UK; Kellgren Centre for Rheumatology, Manchester Royal Infirmary, Manchester University Hospitals NHS Trust, Manchester, UK; Division of Musculoskeletal Medicine and Dermatological Sciences, Centre for Epidemiology Versus Arthritis, University of Manchester, Manchester Academic Health Sciences Centre, Manchester, UK; NIHR Manchester Biomedical Research Centre, Manchester University NHS Foundation Trust, Manchester, UK; Kellgren Centre for Rheumatology, Manchester Royal Infirmary, Manchester University Hospitals NHS Trust, Manchester, UK

**Keywords:** JIA, malignancy, CPRD, epidemiology

## Abstract

**Objectives:**

JIA affects ∼1–2/2000 children in the UK. Adults with inflammatory arthritis have ∼10% increased malignancy risk. JIA malignancy rates in Nordic data are 3–5/10 000 person years, with conflicting evidence on comparative risk. This study calculated malignancy rates in England of patients with JIA, vs matched controls and general population estimates.

**Methods:**

Using UK Primary Care data (CPRD) Aurum, this retrospective cohort study matched patients with JIA (diagnosed <16 years) 4-to-1 with non-JIA controls in England by birth year, gender and practice. Malignancies were identified from (i) NHS-linked hospitalization data and (ii) CPRD read codes. Exposure started at first JIA code date (or matched date for controls), 1 January 2000, or CPRD entry date, whichever was latest. Follow-up continued until end of follow-up of JIA-matched patient (for controls), end of CPRD follow-up, 31 December 2018, first malignancy or death, whichever was first. Cox-proportional hazards models compared malignancy rates. Standardized incidence ratios (SIRs) were calculated against ONS general population estimates by calendar year, age and gender.

**Results:**

A total of 3714 patients with JIA and 10 858 matched controls were identified; demographics were similar. Malignancy rates were 6.8 per 10 000 (95% CI: 4.4–10.7) in JIA and 3.1 per 10 000 person years (95% CI: 2.0–4.6) in controls; HR 2.2 (95% CI: 1.2–4.1). Both cohorts had raised SIRs compared with ONS general population estimates.

**Conclusions:**

This study found that those with JIA had a doubling risk for malignancies compared with the matched control population. However, it is important to note that malignancy in this JIA patient population is extremely rare with the absolute risk remaining very low.

Rheumatology key messages

- Malignancy rates in children, young people and adults with JIA were 6.8 per 10 000 person years.
- Those with JIA had a doubling risk of malignancy compared with the non-JIA control cohort.
- Malignancy in this JIA patient population is extremely rare; the absolute risk remains very low.

## Introduction

JIA is an auto-immune condition that starts in childhood; diagnosed before the age of 16 years old. It affects ∼1–2 in every 2000 children and young people. It is a heterogeneous condition consisting of seven International League of Associations for Rheumatology (ILAR) categories with varying disease characteristics, including but not limited to arthritis, fever, rash, enthesitis, psoriasis and uveitis [1].

There have been persistent concerns about the risk of malignancies in people with inflammatory arthritis, either through the inherent nature of the condition, or the from the treatments being given. A meta-analysis found that adults with RA have a 10% increase in overall malignancy risk compared with the general population [2]. A national RA cohort study found a malignancy rate of 137 per 10 000 person years for those treated with DMARDs, a 28% increased risk of cancer compared with the general population [3]. Adult-onset idiopathic inflammatory myopathy, also known as myositis, confers an increased cancer risk in the 3 years preceding and 3 years after diagnosis [4].

In contrast, malignancy rates in children and young people with JIA have been found to be lower, with reported incidence ranging from 3.3 [5] to 5.0 [6] per 10 000 person years. However, rates have been reported as slightly higher in those treated with biologic therapy; ranging from 5.0 [7] to 9.1 [8] in 10 000 person years. Rates were even higher when looking at adults with JIA starting a TNF inhibitor for the first time in adulthood, 37 per 10 000 person years [9], although this is now an outdated group of individuals as most young people with JIA needing biologic treatment would receive biologic therapy in childhood. Whilst the recent Ehrström *et al.* [6] study did include a median of 10.5 years of follow-up for patients with JIA, it included people with a diagnosis from 1980 which would have also included many individuals diagnosed in the pre-biologics era [6]. Very few other studies have investigated the long-term risk of malignancies, with none in the UK, nor in national databases of more recently diagnosed individuals with JIA.

The aim of this analysis is to calculate the malignancy rate of children, young people and young adults with JIA in the England compared with (i) matched control patients without JIA and (ii) the general population.

## Methods

### Data source

This analysis used data from the UK Clinical Practice Research Datalink (CPRD) Aurum; electronic routine clinical data from primary practitioner practices that use the EMIS software, from ∼7% to 13% of the UK population [10, 11]. In addition, CPRD data were linked to the NHS England Hospital Episode Statistics (HES) data for inpatient admissions and outpatient clinics at hospitals in England. The study was approved by the CPRD Independent Scientific Advisory Committee (protocol number 19_060). CPRD has pre-existing ethical approval from a National Research Ethics Service Committee covering observational research using anonymized CPRD data.

### Patient inclusion

Both prevalent and incident cases of JIA were identified from CPRD Aurum using a pre-defined Read code list [12], that identified all potential cases of arthritis diagnosed in childhood, validated if they met one of two criteria using the NHS HES-linked data; (1) visiting a rheumatologist and/or ophthalmologist ≥3 times within 12-months or (2) a hospital admission ICD-10 code ‘juvenile arthritis’. Control patients without JIA were matched 4-to-1 based on birth year, gender and practice, and could be included twice if they matched with two JIA patients. In addition, patients with a reported code for systemic JIA were identified.

### Outcome and exposure

Malignancies were identified through (i) NHS-linked hospitalization data (all malignancies with an ICD-10 code starting with ‘C’; C00-C97), and (ii) CPRD read codes using a pre-defined codelist. As there was no linkage available with the NHS cancer registry (NCRAS), people with a prior cancer could not be identified, and therefore could not be excluded in this analysis. However, considering this is a young cohort, proportion with prior cancer would have been minimal.

All available CPRD time was included to capture the whole JIA journey. Exposure for each patient started on the date of their first JIA code (or matched date for control patients), 1 January 2000, or their CPRD entry date, whichever was most recent. All patients were followed until the end of their patient follow-up in CPRD, CPRD data cut-off date (31 December 2018), the first reported malignancy or death, whichever was first. Control patients were additionally censored when follow-up ended for their JIA-matched patient. This was to ensure that follow-up in the control population was not overestimated, particularly important when comparing rates between cohorts.

To compare malignancy rates with those that you would have expected in the general population, data from the Office for National Statistics (ONS) were used [13]. Malignancy rates per 100 000 for both males and females were identified for the years 2000–2017 in 5-year age categories; under 1 years old, from 1 to 4 years, from 5 to 9 years, from 10 to 14 years, from 15 to 19 years, from 20 to 24 years, from 25 to 29 years and from 30 to 34 years old. No patients had follow-up over the age of 34 years old. The malignancy rates for the year 2018 were unavailable, therefore the 2017 malignancy rates were used for this calendar year.

### Statistical analysis

Date of birth was calculated using the 15th of the month (imputed as June if missing) and year of birth provided, or start of follow-up (i.e. CPRD entry date) if occurred earlier. Ethnicity, obtained from CPRD observation read codes or HES outpatient and admitted patient care records, was classified using the ONS England ethnic group classification [14]: White, Black, Asian, Mixed or other. Conflicting records were address as per CPRD guidelines [15]. The CPRD Ethnicity Record sources underlying data from Hospital Episode Statistics (HES) Copyright © 2026, re-used with the permission of The Health & Social Care Information Centre. All rights reserved. Index of multiple deprivation (IMD) was obtained for each patient from NHS-linked data and categorized into quintiles, with the 2011 Rural-Urban Classification at LSOA level identified for each GP practice.

ILAR category of JIA is not adequately recorded within CPRD or NHS-linked data. However, within the JIA cohort, patients with a code for systemic JIA were able to identified from CPRD Read codes that included ‘still’ or ‘systemic’ in the arthritis descriptor, or from HES outpatient or admitted patient care diagnoses using the ICD-codes M08.2 (‘Juvenile arthritis with systemic onset’) or M06.1 (‘Adult-onset Still disease’).

Patient characteristics were presented as numbers (percentages) for categorical data, and median (interquartile range; IQR) for continuous data. Malignancy rates were calculated per 10 000 person years. Cox-proportional hazards model was used to compare malignancy rates in JIA vs matched controls. Standardized incidence ratios (SIRs) were generated to compare rates with the ONS general population estimates, based on calendar year, year of age and gender.

### Patient and public involvement statement

This work has been supported by three patient partners, all co-authors on the article. They have all contributed to the analysis plan, interpretation of results and reviewed a conference abstract and this article prior to publication.

## Results

### Patient characteristics

There were 3714 children and young people with JIA (Table 1) matched with 10 858 children and young people without JIA (controls). Patient characteristics were similar with respect to age at entry into CPRD (median 9 years), age at last available follow-up (median 16 years), gender (57% females), IMD and ethnicity. Of the JIA patients, 12% had a recorded code for systemic JIA.

**Table 1:** Patient characteristics of children, young people and young adults with JIA and matched controls.

	Patients with JIA	Control patients
Total patients	3714	10 858
Age at entry to follow-up (years)	9 (5–13)	9 (5–13)
Age at last available follow-up (years)	16 (11–21)	16 (11–19)
Gender		
Male	1586 (43%)	4661 (43%)
Female	2128 (57%)	6197 (57%)
Systemic JIA code		
Yes	434 (12%)	–
No	3280 (88%)	–
Index of multiple deprivation (IMD) quintile	*N* = 3712	*N* = 10 849
Least deprived—*Q*1	796 (21%)	2457 (23%)
*Q*2	725 (20%)	2065 (19%)
*Q*3	692 (19%)	1986 (18%)
*Q*4	711 (19%)	2074 (19%)
Most deprived—*Q*5	788 (21%)	2267 (21%)
Area of practice		
Urban	3258 (88%)	9540 (88%)
Rural	456 (12%)	1318 (12%)
Ethnicity	*N* = 3621	*N* = 8566
White	3081 (85%)	7349 (86%)
Black	150 (4%)	338 (4%)
Asian	273 (8%)	561 (7%)
Mixed	102 (3%)	206 (2%)
Other	15 (<1%)	112 (1%)

Results expressed as number (percentage), or median (interquartile range).

### Malignancy rates

There were 19 malignancies reported over 27 767 years of follow-up in patients with JIA (median follow-up 6.2 years per patient; Table 2); malignancy rate was 6.8 per 10 000 person years (95% CI 4.4–10.7). There were 23 malignancies reported over 74 840 years of follow-up in the control patients (median 5.7 years per patients); malignancy rate 3.1 per 10 000 person years (95% CI 2.0–4.6). Patients with JIA had a doubling in risk for malignancies compared with the control population; hazard ratio 2.2 (95% CI 1.2–4.1). Control patients appeared to be older (median age 24 years old) at the time of malignancy compared with the JIA cohort (median age 16 years).

**Table 2:** Malignancy rates for children, young people and young adults with JIA and matched controls.

	Patients with JIA	Control patients
Total patients	3714	10 858
Total person years	27 767	74 840
Per patient (years)	6.2 (2.6–11.7)	5.7 (2.5–10.4)
Number of patients with first cancer	19	23
Age at entry to follow-up (years)	11 (7–13)	11 (6–14)
Age at first cancer (years)	16 (11–22)	24 (14–28)
Available follow-up (years)	8 (4–17)	17 (5–23)
Gender		
Male	10 (53%)	11 (48%)
Female	9 (47%)	12 (52%)
Malignancy rate, per 10 000 person years	6.8 (4.4–10.7)	3.1 (2.0–4.6)
Hazard ratio (95% CI)	2.2 (1.2–4.1)	[Reference]
SIR (95% CI)	3.3 (2.1–5.2)	1.6 (1.1–2.5)

Standardized incidence ratio (SIR).

Compared with the general population, both JIA and control cohorts had raised SIRs; 3.3 for JIA (95% CI 2.1–5.2) and 1.6 for controls (95% CI 1.1–2.5). This indicated that whilst the JIA population did have an increased risk of malignancies compared with the control population, the CPRD population as a whole had a higher malignancy rate compared with what you would expect given the calendar year, age and gender of the patients. Numbers of malignancies were too small to compare individual cancer types between cohorts, although the proportion of leukaemia cases appeared higher in the JIA cohort (37% vs 22%).

## Discussion

In this large analysis of children, young people and young adults identified in a large primary care dataset, those with JIA had double the hazard for malignancies compared with the matched control population. In addition, both JIA and control cohorts had higher rates of malignancies compared with expected rates in the general population given the calendar year, age and gender of the cohorts.

There have been two large nationwide studies completed relatively recently reporting malignancy rates in those with JIA. Despite including individuals from as early as 1980, Ehrström *et al.* [6] identified that a similar malignancy rate to the current study for those with JIA of 5 per 10 000 person years (vs 6.8 in the current analysis). Ehrström *et al.* also identified that those with JIA had a 1.4 times higher malignancy rate compared with the reference population, similar to this current analysis where those with JIA had 2.2 times higher malignancy rates compared with the non-JIA control cohort. Whereas Horne *et al.* [5] included individuals from 2001, a more similar analysis period to the current one, and identified a lower malignancy rate of 3.3 per 10 000 person years and no significant difference compared with matched controls. Both Nordic studies were similar to the current analysis, including JIA patients from a median age of 10 years (9 years old in the current analysis) and followed patients into adulthood. However, duration of follow-up was difficult to compare as Horne reported a total of 55 107 person years of follow-up without a median per person, and Ehrström report a median of 10.5 years per person without a total person years of follow-up; current analysis included 27 767 total person years, median 6.2 per person.

In this analysis, the population of non-JIA children, young people and young adults had a higher malignancy rate compared with what you would have expected given the population (using national population estimates). Whilst CPRD Aurum has been shown to be representative of the UK population in terms of overall demographics [11], there is an innate selection bias in that those people who develop symptoms of a condition and present to healthcare services who are more likely to have more serious disease [16]. These are the people who will be seeking medical advice from their GP. Individuals may also be more likely to have pre-existing conditions requiring regular follow-up [17], or mean that families are more mindful to take additional symptoms seriously. Considering that the individuals included in this analysis are a very young population, clinicians may tend to have a lower threshold for investigating serious illness such as cancer given the long-term implications of missing a diagnosis at this stage.

This study has many strengths. It used data from a large, representative, national primary care dataset, and validated codelist to identify people with JIA. Patients with JIA were matched to a non-JIA control cohort based on age, gender and general practice, and the median follow-up for all patients was 8 years providing significant follow-up. In addition, the overall patient population included in this analysis was representative of the general population with respective to socioeconomic position, with equal representation over all quintiles of IMD. Whilst it would have been interesting to investigate malignancy rates by ILAR category, this information was not adequately reported in CPRD. Only those with a report of systemic JIA could be identified and even then, numbers were too small to report systemic JIA specific malignancy rates. A limitation of this analysis was that the dataset was not linked to the national cancer registry (NCRAS). Research has shown that in adult cancer, diagnoses are typically under-reported in CPRD compared with both HES and NCRAS, with ∼71% concordance between HES and NCRAS (depending on cancer type) [18]. It is therefore likely that the results in this current research are an underestimation of the malignancy risk in this young patient population, despite the fact that the ONS malignancy figures are based on the NCRAS data, meaning the gap compared with the overall population estimates could be even wider. Cancer staging information was unavailable, and temporal trends could not be assessed due to lower number of malignancies overall. In addition, DMARD therapies are not routinely or accurately reported within CPRD and therefore this information could not be assessed within this research.

This is the first analysis of UK general practice data to investigate the malignancy rate in children, young people and young adults with JIA in England. The mechanism behind the raised malignancy rate in the control cohort compared with the general population requires further investigation, as it could indicate a population with enhanced surveillance, immune related comorbidities or other risk factors. Overall, those with JIA had a doubling risk for malignancies compared with the matched control population. However, it is important to note that malignancy in this JIA patient population is extremely rare with the absolute risk remaining very low.

## Data Availability

The data used in this study were sourced from the CPRD, as part of CPRD protocol 19_060. This protocol can be found on their website (https://cprd.com) where an application can be made to access the data.

## Presented at

This initial results of this research were presented at the British Society for Rheumatology (BSR) annual conference (28–30 April 2026, Glasgow, UK).
